# Supplementary material for: Nanophotonics for pair production
Source: Nat Commun. 2023 Dec 11;14:8189. doi: 10.1038/s41467-023-43701-5 (PMC10713568; doi:10.1038/s41467-023-43701-5)
Supplement: Supplementary file 1 — Supplementary Information [file 41467_2023_43701_MOESM1_ESM.pdf]

Nanophotonics for pair production

– SUPPLEMENTARY INFORMATION –

Valerio Di Giulio[1] and F. Javier García de Abajo[1,2,*]

[1] *ICFO-Institut de Ciencies Fotoniques, The Barcelona Institute of Science and Technology, 08860 Castelldefels (Barcelona), Spain*

[2] *ICREA-Institució Catalana de Recerca i Estudis Avançats, Passeig Lluís Companys 23, 08010 Barcelona, Spain*

(Dated: October 20, 2023)

Contents

Note 1. Pair-production matrix elements for polychromatic classical electromagnetic fields 1
A. Time-dependent perturbation theory 1
B. QED Hamiltonian and matrix elements 2
C. Pair-production rate in second-order perturbation theory 3
D. Alternative derivation of the pair-production rate in the interaction picture 4

Note 2. Pair production by γ-ray interaction with a general polaritonic field 5

Note 3. Pair production by γ-ray interaction with a polaritonic gap mode 6

Note 4. Pair production by γ-ray interaction with a surface polariton 6
A. Pair production close to the threshold 7
B. Pair production with GeV γ-photons 8

Note 5. Additional remarks 9

Supplementary References 9

Note 1. PAIR-PRODUCTION MATRIX ELEMENTS FOR POLYCHROMATIC CLASSICAL ELECTROMAGNETIC FIELDS

To be tutorial for researchers in fields including nanophotonics and quantum optics, we provide a detailed derivation of the pair-production cross section based on standard second-order perturbation theory. We supplement this calculation in Note 1 D below by following the quantum field theory formalism of quantum electrodynamics (QED) [1], more commonly used in the high-energy physics community.

A. Time-dependent perturbation theory

Before specifying the calculation for pair production, we review a general formalism for perturbation theory. A complete set of eigenstates $|j\rangle$ of the unperturbed Hamiltonian $\mathcal{H}_0$ is considered, characterized by energies $\hbar\varepsilon_j$ and, therefore, satisfying $\mathcal{H}_0\,|j\rangle = \hbar\varepsilon_j\,|j\rangle$. Such eigenstates will later be identified with electron and positron number states. We also introduce the perturbation produced by a time-dependent Hamiltonian $\mathcal{H}_{\rm int}(t)$ of matrix elements $\langle j|\,\mathcal{H}_{\rm int}(t)\,|j'\rangle = \sum_i \big(V^i_{jj'}{\rm e}^{-{\rm i}\omega_i t} + V^{i*}_{j'j}{\rm e}^{{\rm i}\omega_i t}\big)$, where the i sum runs over components evolving with frequencies ω_i (corresponding to the interaction with classical polariton and γ-ray fields in the present study). We now expand the time-dependent state of the system as $|\psi(t)\rangle = \sum_j \alpha_j(t)\,{\rm e}^{-{\rm i}\varepsilon_j t}\,|j\rangle$, whose evolution is ruled by the Schrödinger equation $[\mathcal{H}_0 + \mathcal{H}_{\rm int}(t)]\,|\psi(t)\rangle = {\rm i}\hbar\partial_t\,|\psi(t)\rangle$ or, equivalently, the equation of motion

$$\dot\alpha_j(t) = -\frac{\rm i}{\hbar}\sum_{ij'}\big(V^i_{jj'}\,{\rm e}^{-{\rm i}\omega_i t} + V^{i*}_{j'j}\,{\rm e}^{{\rm i}\omega_i t}\big)\,{\rm e}^{{\rm i}\varepsilon_{jj'}t}\,\alpha_{j'}(t)$$

for the expansion coefficients, where we use the notation $\varepsilon_{jj'} = \varepsilon_j - \varepsilon_{j'}$.

*Electronic address: javier.garciadeabajo@nanophotonics.es

Starting from the nondegenerate ground state $|j=0\rangle$ at $t=-\infty$ (the fermionic vacuum in this work), we write the perturbation series $\alpha_j(t)=\sum_n \alpha_j^{(n)}(t)$, where n is the order of interaction and $\alpha_j^{(0)}=\delta_{j0}$ describes the unperturbed state. The first- and second-order terms can be readily obtained upon direct integration as

$$\alpha_j^{(1)}(t) = -\frac{1}{\hbar}\sum_i \left[\frac{V_{j0}^i\,\mathrm{e}^{\mathrm{i}(\varepsilon_{j0}-\omega_i-\mathrm{i}\delta)t}}{\varepsilon_{j0}-\omega_i-\mathrm{i}\delta}+\frac{V_{0j}^{i*}\,\mathrm{e}^{\mathrm{i}(\varepsilon_{j0}+\omega_i-\mathrm{i}\delta)t}}{\varepsilon_{j0}+\omega_i-\mathrm{i}\delta}\right],$$

$$\alpha_j^{(2)}(t) = \frac{1}{\hbar^2}\sum_{ii'j'}\left[\frac{\mathrm{e}^{\mathrm{i}(\varepsilon_{j0}-\omega_i-\omega_{i'}-\mathrm{i}\delta)t}}{(\varepsilon_{j0}-\omega_i-\omega_{i'}-\mathrm{i}\delta)}\frac{V_{jj'}^{i}V_{j'0}^{i'}}{(\varepsilon_{j'0}-\omega_{i'}-\mathrm{i}\delta)}+\frac{\mathrm{e}^{\mathrm{i}(\varepsilon_{j0}+\omega_i+\omega_{i'}-\mathrm{i}\delta)t}}{(\varepsilon_{j0}+\omega_i+\omega_{i'}-\mathrm{i}\delta)}\frac{V_{j'j}^{i*}V_{0j'}^{i'*}}{(\varepsilon_{j'0}+\omega_{i'}-\mathrm{i}\delta)}\right.$$
$$\left.+\frac{\mathrm{e}^{\mathrm{i}(\varepsilon_{j0}-\omega_i+\omega_{i'}-\mathrm{i}\delta)t}}{(\varepsilon_{j0}-\omega_i+\omega_{i'}-\mathrm{i}\delta)}\left(\frac{V_{jj'}^{i}V_{0j'}^{i'*}}{\varepsilon_{j'0}+\omega_{i'}-\mathrm{i}\delta}+\frac{V_{j'j}^{i'*}V_{j'0}^{i}}{\varepsilon_{j'0}-\omega_i-\mathrm{i}\delta}\right)\right],$$

where δ is a positive infinitesimal introduced to adiabatically switch on the interaction. The transition rate at a finite time t is then given by $\Gamma_{0\to j}=\lim_{\delta\to 0^+} d|\alpha_j(t)|^2/dt$. In particular, within first-order perturbation theory (i.e., retaining terms up to order $n=1$ in the perturbation series), we find

$$\Gamma_{0\to j}^{(1)} = \frac{2\pi}{\hbar^2}\sum_i \left|V_{j0}^i\right|^2\delta(\varepsilon_{j0}-\omega_i). \tag{1a}$$

If $\Gamma_{0\to j}^{(1)}$ vanishes, the next leading contribution to the transition rate comes from $\alpha_j^{(2)}(t)$, which yields

$$\Gamma_{0\to j}^{(2)} = \frac{2\pi}{\hbar^4}\sum_i\left[\left|\sum_{j'}\frac{V_{jj'}^iV_{j'0}^i}{\varepsilon_{j'0}-\omega_i-\mathrm{i}\delta}\right|^2\delta(\varepsilon_{j0}-2\omega_i)\right.$$
$$+\sum_{i'<i}\left|\sum_{j'}\left(\frac{V_{jj'}^{i}V_{j'0}^{i'}}{\varepsilon_{j'0}-\omega_{i'}-\mathrm{i}\delta}+\frac{V_{jj'}^{i'}V_{j'0}^{i}}{\varepsilon_{j'0}-\omega_i-\mathrm{i}\delta}\right)\right|^2\delta(\varepsilon_{j0}-\omega_i-\omega_{i'})$$
$$\left.+\sum_{i'}\left|\sum_{j'}\left(\frac{V_{jj'}^{i}V_{0j'}^{i'*}}{\varepsilon_{j'0}+\omega_{i'}-\mathrm{i}\delta}+\frac{V_{j'j}^{i'*}V_{j'0}^{i}}{\varepsilon_{j'0}-\omega_i-\mathrm{i}\delta}\right)\right|^2\delta(\varepsilon_{j0}-\omega_i+\omega_{i'})\right]. \tag{1b}$$

In the derivation of Eqs. (1), we have used the fact that $\varepsilon_j>\varepsilon_0$ and assumed nondegenerate frequencies ω_i and frequency differences $\omega_i-\omega_{i'}$ for $i\neq i'$. In addition, the contribution arising from terms containing two negative energies vanishes because they cannot conserve energy.

B. QED Hamiltonian and matrix elements

We study pair production produced by a classical electromagnetic field that is described through the vector potential $\mathbf{A}(\mathbf{r},t)$ in the temporal gauge (i.e., with a vanishing scalar potential [2, 3]). We adopt the minimal-coupling relativistic QED Hamiltonian in the Schrödinger picture [1]

$$\hat{\mathcal{H}}_{\rm int}(t) = -\frac{1}{c}\int d^3\mathbf{r}\,\hat{\mathbf{j}}(\mathbf{r})\cdot\mathbf{A}(\mathbf{r},t), \tag{2}$$

where $\hat{\mathbf{j}}(\mathbf{r},t)=-ec:\overline{\Psi}(\mathbf{r})\vec{\gamma}\hat{\Psi}(\mathbf{r}):$ is the current operator, we define $\overline{\Psi}=\hat{\Psi}^\dagger\gamma^0$, and the notation $:\cdot:$ is used to indicate normal product acting on the fermionic field operators $\hat{\Psi}(\mathbf{r})$ and $\hat{\Psi}^\dagger(\mathbf{r})$. Here, $\vec{\gamma}$ and γ^0 [1] are the spatial and temporal Dirac matrices. The field operator is then expanded as

$$\hat{\Psi}(\mathbf{r}) = \frac{1}{\sqrt{V}}\sum_{\mathbf{q},s}\left(u_{\mathbf{q},s}\hat{c}_{\mathbf{q},s}\mathrm{e}^{\mathrm{i}\mathbf{q}\cdot\mathbf{r}}+v_{\mathbf{q},s}\hat{d}^\dagger_{\mathbf{q},s}\mathrm{e}^{-\mathrm{i}\mathbf{q}\cdot\mathbf{r}}\right),$$

where V is the normalization volume and we introduce the anticommuting annihilation operators $\hat{c}_{\mathbf{q},s}$ and $\hat{d}_{\mathbf{q},s}$ and the corresponding creation operators $\hat{c}^\dagger_{\mathbf{q},s}$ and $\hat{d}^\dagger_{\mathbf{q},s}$ for electron and positron plane waves of wave vector $\mathbf{q}$ and spin s. The associated 4-component electron and positron spinors $u_{\mathbf{q},s}$ and $v_{\mathbf{q},s}$ are chosen to satisfy the equations

$$(\hbar c\vec{\gamma}\cdot\mathbf{q}+m_ec^2\mathcal{I}_4)\,u_{\mathbf{q},s} = \hbar\varepsilon_q\gamma^0u_{\mathbf{q},s}, \tag{3a}$$
$$(\hbar c\vec{\gamma}\cdot\mathbf{q}-m_ec^2\mathcal{I}_4)\,v_{\mathbf{q},s} = \hbar\varepsilon_q\gamma^0v_{\mathbf{q},s}, \tag{3b}$$

subject to the orthonormalization conditions $u^\dagger_{\mathbf{q},s}u_{\mathbf{q},s'}=\delta_{s,s'}$, $v^\dagger_{\mathbf{q},s}v_{\mathbf{q},s'}=\delta_{s,s'}$ and $u^\dagger_{\mathbf{q},s}v_{-\mathbf{q},s'}=0$. Here, m_e is the electron/positron mass, $\hbar\varepsilon_q=c\sqrt{m_e^2c^2+\hbar^2q^2}$ is the relativistic particle energy, and $\mathcal{I}_4$ is the 4×4 identity matrix.

The current operator takes the explicit form

$$\hat{\mathbf{j}}(\mathbf{r})=-\frac{ec}{V}\sum_{\mathbf{q}\mathbf{q}'}\sum_{ss'}\Big[\hat{c}^\dagger_{\mathbf{q}s}\hat{c}_{\mathbf{q}'s'}\,\mathrm{e}^{\mathrm{i}(\mathbf{q}'-\mathbf{q})\cdot\mathbf{r}}\,\overline{u}_{\mathbf{q}s}\vec{\gamma}\,u_{\mathbf{q}'s'}-\hat{d}^\dagger_{\mathbf{q}s}\hat{d}_{\mathbf{q}'s'}\,\mathrm{e}^{\mathrm{i}(\mathbf{q}'-\mathbf{q})\cdot\mathbf{r}}\,\overline{v}_{\mathbf{q}'s'}\vec{\gamma}\,v_{\mathbf{q}s}$$
$$-\hat{d}^\dagger_{\mathbf{q}s}\hat{c}^\dagger_{\mathbf{q}'s'}\,\mathrm{e}^{-\mathrm{i}(\mathbf{q}+\mathbf{q}')\cdot\mathbf{r}}\,\overline{u}_{\mathbf{q}'s'}\vec{\gamma}\,v_{\mathbf{q}s}-\hat{c}_{\mathbf{q}'s'}\hat{d}_{\mathbf{q}s}\,\mathrm{e}^{\mathrm{i}(\mathbf{q}+\mathbf{q}')\cdot\mathbf{r}}\,\overline{v}_{\mathbf{q}s}\vec{\gamma}\,u_{\mathbf{q}'s'}\Big],\tag{4}$$

where the first two terms describe electron and positron scattering, while the remaining two terms stand for pair creation and annihilation, respectively. In addition, the electromagnetic field is taken to consist of monochromatic components of frequencies ω_i, such that the vector potential can be written

$$\mathbf{A}(\mathbf{r},t)=-\mathrm{i}\,c\sum_i\frac{1}{\omega_i}\vec{\mathcal{E}}_i(\mathbf{r})\mathrm{e}^{-\mathrm{i}\omega_i t}+\text{c.c.},\tag{5}$$

where $\vec{\mathcal{E}}_i(\mathbf{r})$ are the time-independent amplitudes of the field components.

We are now prepared to evaluate the matrix elements of the interaction Hamiltonian in Eq. (2), entering the rates in Eqs. (1) with the j labels running over electron-positron pairs. We thus multiplex $|j\rangle$ as $|p\mathbf{q}s,e\mathbf{q}'s'\rangle=\hat{d}^\dagger_{\mathbf{q}s}\hat{c}^\dagger_{\mathbf{q}'s'}|0\rangle$, where e and p refer to electrons and positrons, respectively. Using Eqs. (2), (4), and (5), we find

$$\begin{aligned}
V^i_{p\mathbf{q}s,e\mathbf{q}'s';p\mathbf{q}s,e\mathbf{q}''s''}&=-\frac{\mathrm{i}ec}{V\omega_i}\,\overline{u}_{\mathbf{q}'s'}\,\vec{\gamma}\cdot\vec{\mathcal{E}}_{i,\mathbf{q}'-\mathbf{q}''}\,u_{\mathbf{q}''s''}, &&\text{electron scattering}\\
V^i_{p\mathbf{q}'s',e\mathbf{q}s;p\mathbf{q}''s'',e\mathbf{q}s}&=+\frac{\mathrm{i}ec}{V\omega_i}\,\overline{v}_{\mathbf{q}''s''}\,\vec{\gamma}\cdot\vec{\mathcal{E}}_{i,\mathbf{q}'-\mathbf{q}''}\,v_{\mathbf{q}'s'}, &&\text{positron scattering}\\
V^i_{p\mathbf{q}s,e\mathbf{q}'s';0}&=+\frac{\mathrm{i}ec}{V\omega_i}\,\overline{u}_{\mathbf{q}'s'}\,\vec{\gamma}\cdot\vec{\mathcal{E}}_{i,\mathbf{q}+\mathbf{q}'}\,v_{\mathbf{q}s}, &&\text{pair creation}\\
V^i_{0;p\mathbf{q}s,e\mathbf{q}'s'}&=+\frac{\mathrm{i}ec}{V\omega_i}\,\overline{v}_{\mathbf{q}s}\,\vec{\gamma}\cdot\vec{\mathcal{E}}_{i,-\mathbf{q}-\mathbf{q}'}\,u_{\mathbf{q}'s'}, &&\text{pair annihilation}
\end{aligned}$$

where

$$\vec{\mathcal{E}}_{i,\mathbf{k}}=\int d^3\mathbf{r}\;\vec{\mathcal{E}}_i(\mathbf{r})\mathrm{e}^{-\mathrm{i}\mathbf{k}\cdot\mathbf{r}}\tag{6}$$

is the Fourier transform of the field amplitudes, which imposes momentum conservation.

C. Pair-production rate in second-order perturbation theory

Pair creation by a single photon is kinematically forbidden [i.e., $\Gamma^{(1)}_{0\to j}=0$], and thus, we need to go to the second order in the light-matter interaction, for which we use Eq. (1b). In the evaluation of $\Gamma^{(2)}_{0\to j}$, it is convenient to analytically carry out the internal sums over j'. Taking the final product as $j\to p\mathbf{q}s,e\mathbf{q}'s'$, the sums in the first and second lines of Eq. (1b) can be evaluated as follows:

$$\begin{aligned}
&\sum_{j'}\left(\frac{V^i_{jj'}V^{i'}_{j'0}}{\varepsilon_{j'0}-\omega_{i'}-\mathrm{i}\delta}+\frac{V^{i'}_{jj'}V^i_{j'0}}{\varepsilon_{j'0}-\omega_i-\mathrm{i}\delta}\right)\\
&=\sum_{\mathbf{q}''s''}\Bigg[\frac{V^i_{p\mathbf{q}s,e\mathbf{q}'s';p\mathbf{q}s,e\mathbf{q}''s''}V^{i'}_{p\mathbf{q}s,e\mathbf{q}''s'';0}}{\varepsilon_{q''}+\varepsilon_q-\omega_{i'}-\mathrm{i}\delta}+\frac{V^i_{p\mathbf{q}s,e\mathbf{q}'s';p\mathbf{q}''s'',e\mathbf{q}'s'}V^{i'}_{p\mathbf{q}''s'',e\mathbf{q}'s';0}}{\varepsilon_{q'}+\varepsilon_{q''}-\omega_{i'}-\mathrm{i}\delta}\\
&\qquad+\frac{V^{i'}_{p\mathbf{q}s,e\mathbf{q}'s';p\mathbf{q}s,e\mathbf{q}''s''}V^i_{p\mathbf{q}s,e\mathbf{q}''s'';0}}{\varepsilon_{q''}+\varepsilon_q-\omega_i-\mathrm{i}\delta}+\frac{V^{i'}_{p\mathbf{q}s,e\mathbf{q}'s';p\mathbf{q}''s'',e\mathbf{q}'s'}V^i_{p\mathbf{q}''s'',e\mathbf{q}'s';0}}{\varepsilon_{q'}+\varepsilon_{q''}-\omega_i-\mathrm{i}\delta}\Bigg]\\
&=\frac{e^2c^2}{V^2\omega_i\omega_{i'}}\sum_{\mathbf{q}''s''}\Bigg[\frac{\overline{u}_{\mathbf{q}'s'}\,\vec{\gamma}\cdot\vec{\mathcal{E}}_{i,\mathbf{q}'-\mathbf{q}''}\,u_{\mathbf{q}''s''}\,\overline{u}_{\mathbf{q}''s''}\,\vec{\gamma}\cdot\vec{\mathcal{E}}_{i',\mathbf{q}+\mathbf{q}''}\,v_{\mathbf{q}s}}{\varepsilon_{q''}-(\varepsilon_{q'}-\omega_i)-\mathrm{i}\delta}-\frac{\overline{v}_{\mathbf{q}''s''}\,\vec{\gamma}\cdot\vec{\mathcal{E}}_{i,\mathbf{q}-\mathbf{q}''}\,v_{\mathbf{q}s}\,\overline{u}_{\mathbf{q}'s'}\,\vec{\gamma}\cdot\vec{\mathcal{E}}_{i',\mathbf{q}'+\mathbf{q}''}\,v_{\mathbf{q}''s''}}{\varepsilon_{q''}+(\varepsilon_{q'}-\omega_{i'})-\mathrm{i}\delta}\\
&\qquad+\frac{\overline{u}_{\mathbf{q}'s'}\,\vec{\gamma}\cdot\vec{\mathcal{E}}_{i',\mathbf{q}'-\mathbf{q}''}\,u_{\mathbf{q}''s''}\,\overline{u}_{\mathbf{q}''s''}\,\vec{\gamma}\cdot\vec{\mathcal{E}}_{i,\mathbf{q}+\mathbf{q}''}\,v_{\mathbf{q}s}}{\varepsilon_{q''}-(\varepsilon_{q'}-\omega_{i'})-\mathrm{i}\delta}-\frac{\overline{v}_{\mathbf{q}''s''}\,\vec{\gamma}\cdot\vec{\mathcal{E}}_{i',\mathbf{q}-\mathbf{q}''}\,v_{\mathbf{q}s}\,\overline{u}_{\mathbf{q}'s'}\,\vec{\gamma}\cdot\vec{\mathcal{E}}_{i,\mathbf{q}'+\mathbf{q}''}\,v_{\mathbf{q}''s''}}{\varepsilon_{q''}+(\varepsilon_{q'}-\omega_i)-\mathrm{i}\delta}\Bigg]\\
&=-\frac{e^2c^2}{V^2\omega_i\omega_{i'}}\overline{u}_{\mathbf{q}'s'}\,\vec{\gamma}\cdot\sum_{\mathbf{q}''}\Big[\vec{\mathcal{E}}_{i,\mathbf{q}'-\mathbf{q}''}\,G_F(\mathbf{q}'',\varepsilon_{q'}-\omega_i)\,\vec{\mathcal{E}}_{i',\mathbf{q}+\mathbf{q}''}+\vec{\mathcal{E}}_{i',\mathbf{q}'-\mathbf{q}''}\,G_F(\mathbf{q}'',\varepsilon_{q'}-\omega_{i'})\,\vec{\mathcal{E}}_{i,\mathbf{q}+\mathbf{q}''}\Big]\cdot\vec{\gamma}\,v_{\mathbf{q}s},
\end{aligned}\tag{7}$$

where

$$G_F(\mathbf{q},\omega) = -\sum_s \left(\frac{u_{\mathbf{q}s}\,\overline{u}_{\mathbf{q}s}}{\varepsilon_q-\omega-\mathrm{i}\delta} - \frac{v_{-\mathbf{q}s}\,\overline{v}_{-\mathbf{q}s}}{\varepsilon_q+\omega-\mathrm{i}\delta}\right) = \frac{\omega\gamma^0 - c\vec{\gamma}\cdot\mathbf{q} + (m_e c^2/\hbar)\,\mathcal{I}_4}{\omega^2-\varepsilon_q^2+\mathrm{i}0^+}. \tag{8}$$

is the so-called Feynman propagator [1]. In the derivation of this result, we have invoked energy conservation [i.e., the condition $\varepsilon_q+\varepsilon_{q'}=\omega_i+\omega_{i'}$ imposed by the δ-function in Eq. (1b)] and changed $\mathbf{q}''\to-\mathbf{q}''$ in the positron-scattering terms. In addition, the second line of Eq. (8) is obtained from the first one by first combining the two fractions and then using Eqs. (3) to eliminate ε_q in the numerator, applying the completeness relation $\sum_s \left(u_{\mathbf{q}s}\,u^\dagger_{\mathbf{q}s} + v_{-\mathbf{q}s}\,v^\dagger_{-\mathbf{q}s}\right)=\mathcal{I}_4$, and taking the $\delta\to0^+$ limit. Following a similar procedure and making use of the identity $(\overline{u}\,\vec{\gamma}\,v)^* = -\overline{v}\,\vec{\gamma}\,u$, we find

$$\sum_{j'}\left(\frac{V^i_{jj'}V^{i'*}_{0j'}}{\varepsilon_{j'0}+\omega_{i'}-\mathrm{i}\delta} + \frac{V^{i'*}_{j'j}V^i_{j'0}}{\varepsilon_{j'0}-\omega_i-\mathrm{i}\delta}\right) = \frac{e^2c^2}{V^2\omega_i\omega_{i'}}\overline{u}_{\mathbf{q}'s'}\,\vec{\gamma}\cdot\sum_{\mathbf{q}''}\left[\vec{\mathcal{E}}_{i,\mathbf{q}'-\mathbf{q}''}\,G_F(\mathbf{q}'',\varepsilon_{q'}-\omega_i)\,\vec{\mathcal{E}}^*_{i',-\mathbf{q}-\mathbf{q}''} + \vec{\mathcal{E}}^*_{i',\mathbf{q}''-\mathbf{q}'}\,G_F(\mathbf{q}'',\varepsilon_{q'}+\omega_{i'})\,\vec{\mathcal{E}}_{i,\mathbf{q}+\mathbf{q}''}\right]\cdot\vec{\gamma}\,v_{\mathbf{q}s} \tag{9}$$

for the j' sum in the third line of Eq. (1b).

Finally, using Eqs. (7) and (9) in Eq. (1b) and ignoring contributions from two photons of the same frequency (because we are interested in polariton and γ-ray scattering), we find the second-order pair-production rate

$$\Gamma^{(2)}_{p\mathbf{q}s,e\mathbf{q}'s'} = \frac{2\pi e^4c^4}{V^4\hbar^4}\sum_{ii'}{}'\frac{1}{\omega_i^2\omega_{i'}^2}\sum_{\pm}\delta(\varepsilon_q+\varepsilon_{q'}-\omega_i\pm\omega_{i'}) \times\left|\overline{u}_{\mathbf{q}'s'}\,\vec{\gamma}\cdot\sum_{\mathbf{q}''}\left[\vec{\mathcal{E}}_{i,\mathbf{q}'-\mathbf{q}''}\,G_F(\mathbf{q}'',\varepsilon_{q'}-\omega_i)\,\vec{\mathcal{E}}^{\pm}_{i',\mathbf{q}+\mathbf{q}''} + \vec{\mathcal{E}}^{\pm}_{i',\mathbf{q}'-\mathbf{q}''}\,G_F(\mathbf{q}'',\varepsilon_{q'}\pm\omega_{i'})\,\vec{\mathcal{E}}_{i,\mathbf{q}+\mathbf{q}''}\right]\cdot\vec{\gamma}\,v_{\mathbf{q}s}\right|^2, \tag{10}$$

where we have defined $\vec{\mathcal{E}}^+_{i',\mathbf{k}}\equiv\vec{\mathcal{E}}^*_{i',-\mathbf{k}}$ and $\vec{\mathcal{E}}^-_{i',\mathbf{k}}\equiv\vec{\mathcal{E}}_{i',\mathbf{k}}$, while the prime in the summation symbol indicates that it is restricted to $\omega_{i'}<\omega_i$. Equation (10) describes pair production (an electron of wave vector $\mathbf{q}'$ and spin s', combined with a positron of wave vector $\mathbf{q}$ and spin s) for any arbitrary field comprising components of nondegenerate frequencies ω_i.

D. Alternative derivation of the pair-production rate in the interaction picture

An alternative procedure to calculate the desired production rate consists in starting from the interaction Hamiltonian in the interaction picture $\hat{\mathcal{H}}^I_{\rm int}(t)=\mathrm{e}^{\mathrm{i}\hat{\mathcal{H}}_0t/\hbar}\hat{\mathcal{H}}\mathrm{e}^{-\mathrm{i}\hat{\mathcal{H}}_0t/\hbar}$. We are interested in obtaining the leading contribution to the probability amplitude connecting the initial fermionic vacuum state $|0\rangle$ to a final pair state $|p\mathbf{q}s,e\mathbf{q}'s'\rangle$, which we write as $C_{p\mathbf{q}s,e\mathbf{q}'s'}=\langle p\mathbf{q}s,e\mathbf{q}'s'|\,\hat{\mathcal{S}}(\infty,-\infty)\,|0\rangle$ in terms of the scattering operator $\hat{\mathcal{S}}(t_2,t_1)=\mathcal{T}\,\mathrm{e}^{-(\mathrm{i}/\hbar)\int_{t_1}^{t_2}dt\;\hat{\mathcal{H}}^I_{\rm int}(t)}$, where $\mathcal{T}$ stands for the time ordering operator. By retaining only quadratic terms in the electromagnetic field and working out time ordering through Wick's theorem [1], we obtain

$$C_{p\mathbf{q}s,e\mathbf{q}'s'}\approx\frac{-\mathrm{i}e^2}{\hbar^2}\int_{-\infty}^{\infty}dt\int_{-\infty}^{\infty}dt'\int d^3\mathbf{r}\int d^3\mathbf{r}'\;\langle p\mathbf{q}s,e\mathbf{q}'s'|:\overline{\Psi}(\mathbf{r},t)\vec{\gamma}\cdot\mathbf{A}(\mathbf{r},t)G_F(\mathbf{r}-\mathbf{r}',t-t')\vec{\gamma}\cdot\mathbf{A}(\mathbf{r}',t')\hat{\Psi}(\mathbf{r}',t'):|0\rangle\,,$$

where $G_F(\mathbf{r},t)=(2\pi)^{-4}\int_{-\infty}^{\infty}d\omega\int d^3\mathbf{q}\,\mathrm{e}^{\mathrm{i}\mathbf{q}\cdot\mathbf{r}-\mathrm{i}\omega t}G_F(\mathbf{q},\omega)$ is the real-spacetime representation of the Feynman propagator defined in Eq. (8). Plugging the vector potential defined in Eq. (5) and carrying out the required Dirac matrix algebra, this expression reduces to

$$C^{\pm}_{p\mathbf{q}s,e\mathbf{q}'s'}\approx\frac{2\pi\mathrm{i}e^2c^2}{V^2\hbar^2}\sum_{ii'}{}'\frac{1}{\omega_i\omega_{i'}}\,\delta(\varepsilon_q+\varepsilon_{q'}-\omega_i\pm\omega_{i'}) \times\overline{u}_{\mathbf{q}'s'}\,\vec{\gamma}\cdot\sum_{\mathbf{q}''}\left[\vec{\mathcal{E}}_{i,\mathbf{q}'-\mathbf{q}''}\,G_F(\mathbf{q}'',\varepsilon_{q'}-\omega_i)\,\vec{\mathcal{E}}^{\pm}_{i',\mathbf{q}+\mathbf{q}''} + \vec{\mathcal{E}}^{\pm}_{i',\mathbf{q}'-\mathbf{q}''}\,G_F(\mathbf{q}'',\varepsilon_{q'}\pm\omega_{i'})\,\vec{\mathcal{E}}_{i,\mathbf{q}+\mathbf{q}''}\right]\cdot\vec{\gamma}\,v_{\mathbf{q}s},$$

where $\vec{\mathcal{E}}_{i,\mathbf{k}}$ is defined in Eq. (6) and the $\pm$ sign refers to channels involving either two frequencies of opposite sign (+) or two positive frequencies (−). Again, the prime in the summation symbol restricts it to $\omega_{i'}<\omega_i$

terms. Finally, the transition rate is obtained as $\Gamma^{(2)}_{\mathbf{pq}s,e\mathbf{q}'s'} = |C^{\pm}_{\mathbf{pq}s,e\mathbf{q}'s'}|^2/T$, where T is the interaction time. This expression produces a squared δ-function that we need to reinterpret by retaining one of such functions coming from one of the two $C^{\pm}_{\mathbf{pq}s,e\mathbf{q}'s'}$ factors and then undoing the time integral in the other factor through the prescription $\delta \to (2\pi)^{-1}\int dt$; the remaining δ-function still imposes energy conservation, whereas the undone time integral yields a factor T that cancels with the denominator. Following this procedure, we readily find a result that coincides with Eq. (10).

Note 2. PAIR PRODUCTION BY γ-RAY INTERACTION WITH A GENERAL POLARITONIC FIELD

Equation (10) can be generally applied to an arbitrary number of field components. Here, we are interested in calculating the pair-production rate associated with the scattering of surface polaritons of frequency ω_p $(i = p)$ and highly energetic ($> 2m_ec^2 \approx 1.02\,$MeV) γ-ray photons of frequency ω_γ $(i = \gamma)$. We consider a general polaritonic field $\vec{\mathcal{E}}_p(\mathbf{r})$, from which the Fourier-transformed field $\vec{\mathcal{E}}_{p,\mathbf{k}}$ is obtained by using Eq. (6). Likewise, we write $\vec{\mathcal{E}}_\gamma(\mathbf{r}) = E_\gamma\,\hat{\mathbf{e}}_j\,\mathrm{e}^{\mathrm{i}\mathbf{k}_\gamma\cdot\mathbf{r}}$ for a γ-ray plane-wave field of amplitude E_γ, wave vector $\mathbf{k}_\gamma$ (taking along $\hat{\mathbf{z}}$), and unit polarization vector $\hat{\mathbf{e}}_j = \hat{\mathbf{x}}$ or $\hat{\mathbf{y}}$ for $j = 1$ or 2, respectively, leading to $\vec{\mathcal{E}}_{\gamma\mathbf{k}} = V\,E_\gamma\,\hat{\mathbf{e}}_j\,\delta_{\mathbf{k},\mathbf{k}_\gamma}$. We neglect material polarization at the high γ-photon frequency. Inserting these expressions for the momentum-space fields into Eq. (10), and noticing that the only term in the (i,i') sum satisfying $\omega_{i'} < \omega_i$ corresponds to the choice $i = \gamma$ and $i' = p$, we find the rate

$$\Gamma^{(2)}_{\mathbf{pq}s,e\mathbf{q}'s'} = \frac{\pi\alpha^2c^6|E_\gamma|^2}{V^2\hbar^2\omega_\gamma^2\omega_p^2}\sum_{\pm}\delta(\varepsilon_q+\varepsilon_{q'}-\omega_\gamma\pm\omega_p)\sum_{j=1,2}\left|\overline{u}_{\mathbf{q}'s'}\,\mathcal{M}^{\pm}_j(\mathbf{q},\mathbf{q}')\,v_{\mathbf{q}s}\right|^2,$$

where $\alpha \approx 1/137$ is the fine-structure constant, the average over γ-ray polarization is performed (j sum), we recall that primed (unprimed) quantities refer to the electron (positron), and we define the 4×4 matrix

$$\mathcal{M}^{\pm}_j(\mathbf{q},\mathbf{q}') = \gamma^j\,G_F(\mathbf{q}'-\mathbf{k}_\gamma,\varepsilon_{q'}-\omega_\gamma)\,\vec{\gamma}\cdot\vec{\mathcal{E}}^{\pm}_{p,\mathbf{q}+\mathbf{q}'-\mathbf{k}_\gamma} + \vec{\mathcal{E}}^{\pm}_{p,\mathbf{q}+\mathbf{q}'-\mathbf{k}_\gamma}\cdot\vec{\gamma}\,G_F(\mathbf{k}_\gamma-\mathbf{q},\omega_\gamma-\varepsilon_q)\,\gamma^j \tag{11}$$

with G_F given by Eq. (8).

It is convenient to recast this result in the form of a polariton-driven pair-production cross section $\sigma^{\rm pol}_{\mathbf{pq}s,e\mathbf{q}'s'} = \Gamma^{(2)}_{\mathbf{pq}s,e\mathbf{q}'s'}/N_pF_\gamma$, which is calculated by normalizing the rate to both the number of polaritons in the material (N_p) and the γ-photon flux traversing the polariton-supporting interface (F_γ). More precisely, we obtain N_p as the space integral of the field energy density divided by the polariton energy,

$$N_p = \frac{1}{4\pi\hbar\omega_p}\int d^3\mathbf{r}\left\{\partial_{\omega_p}\mathrm{Re}\{\omega_p\epsilon(\mathbf{r},\omega_p)\}\,|\vec{\mathcal{E}}_p(\mathbf{r})|^2 + (c/\omega_p)^2|\nabla\times\vec{\mathcal{E}}_p(\mathbf{r})|^2\right\}, \tag{12}$$

where $\epsilon(\mathbf{r},\omega_p)$ is the position-dependent permittivity of the involved materials at the polariton frequency ω_p. Here, the local response approximation is adopted and polaritons are assumed to be lossless as a reasonable description of long-lived modes. In addition, the γ-photon flux is derived from the associated intensity divided by the photon energy as $F_\gamma = c\,|E_\gamma|^2/2\pi\hbar\omega_\gamma$. Putting these elements together, we find the momentum-resolved positron-emission cross section

$$\begin{aligned}\frac{d\sigma^{\rm pol}}{d\mathbf{q}} &= \frac{V}{(2\pi)^3}\sum_{ss'}\sum_{\mathbf{q}'}\sigma^{\rm pol}_{\mathbf{pq}s,e\mathbf{q}'s'}\\ &= \frac{\alpha^2c^5}{32\pi^4N_p\hbar\omega_\gamma\omega_p^2}\int d^3\mathbf{q}'\sum_{\pm}\delta(\varepsilon_q+\varepsilon_{q'}-\omega_\gamma\pm\omega_p)\sum_{ss'}\sum_{j=1,2}\left|\overline{u}_{\mathbf{q}'s'}\,\mathcal{M}^{\pm}_j(\mathbf{q},\mathbf{q}')\,v_{\mathbf{q}s}\right|^2,\end{aligned} \tag{13}$$

which includes a sum over emitted-particle spins and incorporates the prescription $\sum_{\mathbf{q}} \to (2\pi)^{-3}V\int d^3\mathbf{q}$ to transform sums over particle wave vectors into integrals. The cross section in Eq. (13) is normalized in such a way that the total positron-emission cross section is given by $\sigma^{\rm pol} = \int d^3\mathbf{q}\,(d\sigma^{\rm pol}/d\mathbf{q})$. Finally, the energy- and polar-angle-resolved positron-emission cross section is obtained by integrating Eq. (13) over the azimuthal emission angle φ as

$$\frac{d\sigma^{\rm pol}}{dE_qd\theta} = \sin\theta\,\frac{q\varepsilon_q}{\hbar c^2}\int_0^{2\pi}d\varphi\,\frac{d\sigma^{\rm pol}}{d\mathbf{q}}, \tag{14}$$

where $E_q = \hbar\varepsilon_q$ is the positron energy and θ is the emission angle relative to the z axis.

Note 3. PAIR PRODUCTION BY γ-RAY INTERACTION WITH A POLARITONIC GAP MODE

Equation (13) gives the positron emission cross section for a general polaritonic field. Polaritonic gap modes are particularly interesting because they can enhance the optical field by several orders of magnitude relative to the incident light field within a small spatial region [4]. To estimate the effect of field confinement and enhancement on the pair-production yield, we consider a mode field of uniform amplitude E_p and unit polarization vector $\hat{\mathbf{x}}$ defined within a sphere of radius R_p, such that $\vec{\mathcal{E}}_p(\mathbf{r}) = E_p\hat{\mathbf{x}}\,\Theta(R_p - r)$. The Fourier transform of this field [Eq. (6)] yields

$$\vec{\mathcal{E}}_{p,\mathbf{k}} = \frac{4\pi E_p\,\hat{\mathbf{x}}}{k^3}\left[\sin(kR_p) - kR_p\cos(kR_p)\right]. \tag{15}$$

To compute the cross section per polariton and γ-photon, we plug Eq. (15) into Eq. (13) and divide the result by the number of polaritons, which is obtained from Eq. (12) as $N_p \approx E_p^2 R_p^3/3\hbar\omega_p$ after disregarding the energy contribution from the magnetic field (this part is negligible for highly confined modes supported by the electric polarization of the involved materials). Finally, transforming the δ function in Eq. (13) as $\delta(\varepsilon_q + \varepsilon_{q'} - \omega_\gamma \pm \omega_p) = \delta(q' - q'_\pm)/\partial_{q'}\varepsilon_{q'}$ with $\partial_{q'}\varepsilon_{q'} = c^2q'/\varepsilon_{q'}$, we obtain

$$\begin{aligned}\frac{d\sigma^{\rm pol}}{d\mathbf{q}} =& \frac{3\alpha^2c^3}{2\pi^2\omega_\gamma\omega_pR_p^3}\int d\Omega_{\mathbf{q}'}\sum_{\pm}\left[\sin(k_{p\pm}R_p) - k_{p\pm}R_p\cos(k_{p\pm}R_p)\right]^2\Theta(\omega_\gamma \mp \omega_p - m_ec^2/\hbar - \varepsilon_q) \\ &\times\frac{q'_\pm\varepsilon_{q'_\pm}}{k_{p\pm}^6}\sum_{ss'}\sum_{j=1,2}\left|\overline{u}_{\tilde{\mathbf{q}}'_\pm s'}\left[\gamma^j\,G_F(\mathbf{q}'_\pm - \mathbf{k}_\gamma, \varepsilon_{q'_\pm} - \omega_\gamma)\,\gamma^1 + \gamma^1\,G_F(\mathbf{k}_\gamma - \mathbf{q}, \omega_\gamma - \varepsilon_q)\,\gamma^j\right]v_{\mathbf{q}s}\right|^2,\end{aligned} \tag{16}$$

where $k_{p\pm} = |\mathbf{q} + \mathbf{q}'_\pm - \mathbf{k}_\gamma|$, while $q'_\pm$ must satisfy the energy conservation condition $\varepsilon_{q'_\pm} = \omega_\gamma - \varepsilon_q \mp \omega_p$.

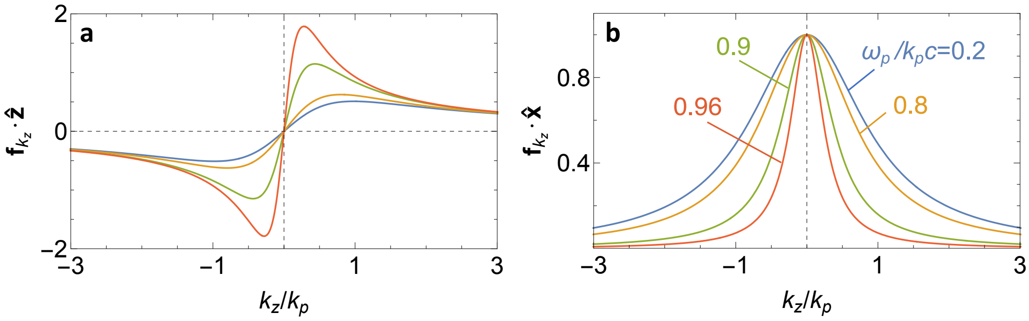

Supplementary Figure 1: Momentum distribution associated with the polariton field. We plot the components of $\mathbf{f}_{k_z}$ [Eq. (18)] along (a) out-of-plane and (b) in-plane directions as a function of k_z (normalized to the polariton wave vector k_p) for various polariton frequencies ω_p (normalized to k_pc).

Note 4. PAIR PRODUCTION BY γ-RAY INTERACTION WITH A SURFACE POLARITON

We consider polaritons bound to a planar material interface of area A placed in the $z = 0$ plane (e.g., a two-dimensional material capable of supporting strongly confined polaritons [5, 6], such as graphene [7], few-atomic-layer hexagonal boron nitride [8], or ultrathin metal films [9]). Polaritons are taken to be lossless and traveling with a real in-plane wave vector $\mathbf{k}_p = k_p\hat{\mathbf{x}}$ (with $k_p > \omega_p/c$) oriented along the x direction, so that their associated electric field can be written as $\vec{\mathcal{E}}_p(\mathbf{r}) = (E_pc/\omega_p)\big(\mathrm{i}\kappa_p\,\hat{\mathbf{x}} - k_p\,\mathrm{sign}\{z\}\,\hat{\mathbf{z}}\big)\mathrm{e}^{\mathrm{i}k_px - \kappa_p|z|}$, where E_p is a global amplitude and $\kappa_p = \sqrt{k_p^2 - \omega_p^2/c^2}$ describes the evanescent field decay away from the interface. The Fourier transform of this field [see Eq. (6)] is

$$\vec{\mathcal{E}}_{p\mathbf{k}} = \frac{2\mathrm{i}A\,c}{\omega_p}\,E_p\,\mathbf{f}_{k_z}\,\delta_{\mathbf{k}_\parallel,\mathbf{k}_p} = \frac{2\mathrm{i}\,c}{\omega_p}\,E_p\,\mathbf{f}_{k_z}\,(2\pi)^2\delta(\mathbf{k}_\parallel - \mathbf{k}_p), \tag{17}$$

where the subscript $\parallel$ denotes the in-plane x-y components, we define the real vector

$$\mathbf{f}_{k_z} = \frac{\kappa_p^2\,\hat{\mathbf{x}} + k_pk_z\,\hat{\mathbf{z}}}{\kappa_p^2 + k_z^2}, \tag{18}$$

and the transformation from the central to the rightmost parts of Eq. (17) is carried out by using the relation $(2\pi)^2\delta(\mathbf{k}_\parallel-\mathbf{k}_p)=A\,\delta_{\mathbf{k}_\parallel,\mathbf{k}_p}$ between the Kroneker and Dirac δ functions. The lack of translational invariance along the out-of-plane direction introduces a finite range of momentum mismatch in that direction relative to the $q_z+q'_z=k_{\gamma z}$ condition, as described by the k_z dependence of $\mathbf{f}_{k_z}$, which we illustrate in Supplementary Figure **1** [see also Eq. (20) below]. From this polaritonic field, Eq. (12) yields a number of polaritons $N_p=A\,|E_p|^2k_p^2c^2/2\pi\hbar\omega_p^3\kappa_p$. Inserting this result together with Eq. (17) in Eq. (13), we find

$$\frac{d\sigma^{\rm pol}}{d\mathbf{q}}=\frac{\alpha^2c^5\kappa_p}{\pi\omega_p\omega_\gamma k_p^2}\int d^3\mathbf{q}'\sum_\pm\,\delta(\mathbf{k}_{\gamma\parallel}-\mathbf{q}_\parallel-\mathbf{q}'_\parallel\mp\mathbf{k}_p)\,\delta(\varepsilon_q+\varepsilon_{q'}-\omega_\gamma\pm\omega_p) \tag{19}$$
$$\times\sum_{ss'}\sum_{j=1,2}\Big|\overline{u}_{\mathbf{q}'s'}\,\mathcal{N}_j^\pm(\mathbf{q},\mathbf{q}')\,v_{\mathbf{q}s}\Big|^2,$$

where the 4×4 matrix

$$\mathcal{N}_j^\pm(\mathbf{q},\mathbf{q}')=\gamma^j\,G_F(\mathbf{q}'-\mathbf{k}_\gamma,\varepsilon_{q'}-\omega_\gamma)\,\vec{\gamma}\cdot\mathbf{f}_{\pm(k_{\gamma z}-q_z-q'_z)}+\mathbf{f}_{\pm(k_{\gamma z}-q_z-q'_z)}\cdot\vec{\gamma}\,G_F(\mathbf{k}_\gamma-\mathbf{q},\varepsilon_{q'}\pm\omega_p)\,\gamma^j \tag{20}$$

is obtained from Eq. (11) upon substitution of $\vec{\mathcal{E}}^\pm_{p,\mathbf{q}+\mathbf{q}'-\mathbf{k}_\gamma}$ by $\mathbf{f}_{\pm(k_{\gamma z}-q_z-q'_z)}$. The latter incorporates the anticipated finite out-of-plane momentum distribution. For a given emitted positron wave vector $\mathbf{q}$, the electron wave vector $\mathbf{q}'$ is determined by the δ functions in Eq. (19). In particular, the in-plane electron wave vector is given by $\mathbf{q}'_{\parallel\pm}=\mathbf{k}_{\gamma\parallel}-\mathbf{q}_\parallel\mp\mathbf{k}_p$. Also, noticing the relation $\partial_{q'_z}\varepsilon_{q'}=q'_zc^2/\varepsilon_{q'}$, we can write

$$\delta(\varepsilon_q+\varepsilon_{q'}-\omega_\gamma\pm\omega_p)=\frac{\varepsilon_{q'_\pm}}{c^2q'_{z\pm}}\,\left[\delta(q'_z-q'_{z\pm})+\delta(q'_z+q'_{z\pm})\right]\,\Theta\Big(\varepsilon^2_{q'_\pm}-m_e^2c^4/\hbar^2-c^2q'^2_{\parallel\pm}\Big)\,\Theta\Big(\omega_\gamma\mp\omega_p-\varepsilon_q\Big),$$

where

$$q'_{z\pm}=\sqrt{\varepsilon^2_{q'_\pm}/c^2-m_e^2c^2/\hbar^2-q'^2_{\parallel\pm}} \tag{21}$$

is the out-of-plane electron wave-vector component and $\varepsilon_{q'_\pm}=\omega_\gamma\mp\omega_p-\varepsilon_q$ is the electron energy. This allows us to recast the wave-vector-resolved differential positron emission cross section as

$$\frac{d\sigma^{\rm pol}}{d\mathbf{q}}=\frac{\alpha^2c^3\kappa_p}{\pi\,\omega_p\omega_\gamma k_p^2}\sum_\pm\frac{\varepsilon_{q'_\pm}}{q'_{z\pm}}\,\Theta\Big(\varepsilon^2_{q'_\pm}-m_e^2c^4/\hbar^2-c^2q'^2_{\parallel\pm}\Big)\,\Theta\Big(\omega_\gamma\mp\omega_p-\varepsilon_q\Big) \tag{22}$$
$$\times\sum_{ss'}\sum_{j=1,2}\sum_{\mu=\pm1}\Big|\overline{u}_{\mathbf{q}'_{\parallel\pm}+\mu q'_{z\pm}\hat{\mathbf{z}},s'}\,\mathcal{N}_j^\pm(\mathbf{q},\mathbf{q}'_{\parallel\pm}+\mu q'_{z\pm}\hat{\mathbf{z}})\,v_{\mathbf{q}s}\Big|^2,$$

with $q'_{z\pm}$ given in Eq. (21), such that the $\mu=1$ and $\mu=-1$ terms stand for the contributions associated with upward ($q'_z=+q'_{z\pm}$) and downward ($q'_z=-q'_{z\pm}$) electron emission, respectively.

Finally, we insert Eq. (22) into Eq. (14) to compute $d\sigma^{\rm pol}/dE_qd\theta$ in Supplementary Figures **2** and **3** below, where we present this quantity after averaging it over a finite positron energy range $\Delta E_q=1$ keV just to make the plot clearer by smoothing the integrable divergence introduced by the $1/q'_{z\pm}$ factor in Eq. (22) at the onset of positron emission.

A. Pair production close to the threshold

From Eq. (22) and the discussion presented in the main text, we expect positron production by mixing polaritons and $\gtrsim1.02$ MeV photons, such as those available from commonly used sources [12, 13] (e.g., ^{60}Co, which emits at ~1.17 MeV and ~1.33 MeV with a lifetime of ~5.13 years, yielding $\sim10^{14}$ photons/s out of 1 g of material).

To put the present results in context, we note that the free-space Breit-Wheeler (BW) cross section (see point 3 of **Note 5**) is very small for pair production out of such γ-photons alone [e.g., the maximum cross section is $\sigma^{\rm BW}\lesssim0.17$ barn (1 barn $=10^{-24}$ cm^2) for two 1.33 MeV photons]. We illustrate this by considering an arrangement consisting of two facing ^{60}Co sources spaced by a few meters so that $\sim10^6$ photons are simultaneously traveling across that distance, and therefore, $\sim10^{12}$ photon-photon collisions take place during the traveling time $\sim10^{-8}$ s. Now, multiplying the number of collisions by $\sigma^{\rm BW}$ and dividing by both a transverse area of ~1 m^2 and the traveling time, we estimate a pair-production rate of $\sim10^{-9}$/s.

Polaritons can be made in large supply over small spatial regions by relying on ultrafast lasers (e.g., one has $\sim10^{19}$ photons in 1 J pulses of 100 fs duration, such as those delivered by tabletop setups, which could be schemed to achieve nearly complete coupling to polaritons [14]). This allows us to partially compensate

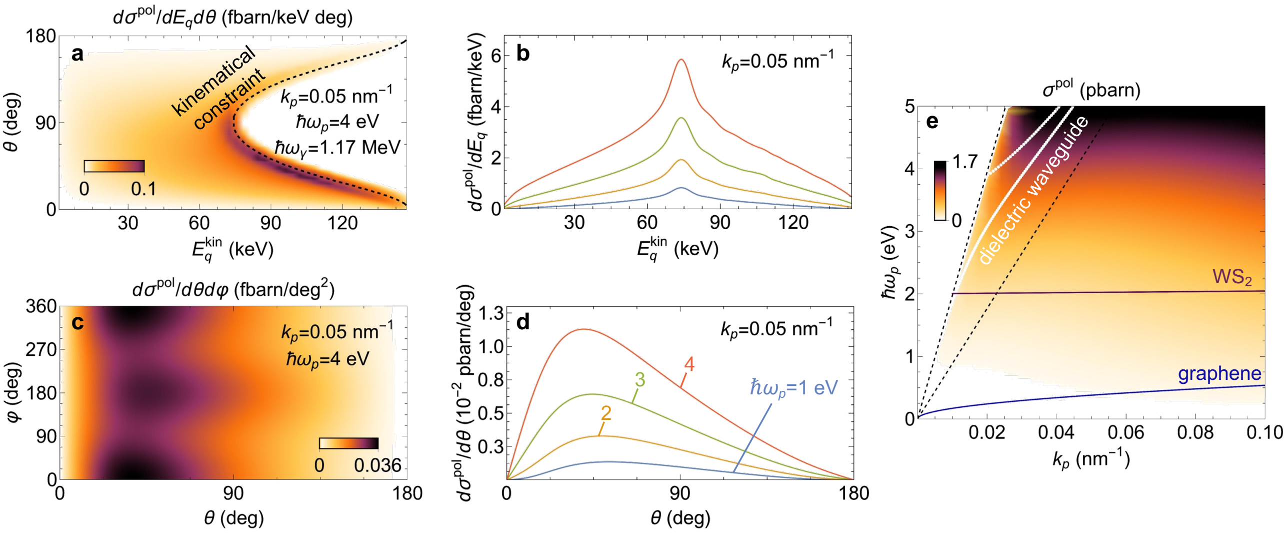

Supplementary Figure 2: **Pair-production cross section near the threshold.** (a) Differential cross section for positron emission as a function of polar angle θ and kinetic energy $E_q^{\rm kin} = \hbar\varepsilon_q - m_e c^2$ (normalized to the γ-photon energy $\hbar\omega_\gamma = 1.17$ MeV and averaged over a window $\Delta E_q^{\rm kin} = 8$ keV) for fixed polariton wave vector $k_p = 0.05$ nm^{-1} and energy $\hbar\omega_p = 4$ eV, as computed from $d\sigma^{\rm pol}/dE_q d\theta = \sin\theta\,(q\varepsilon_q/\hbar c^2)\int_0^{2\pi} d\varphi\;(d\sigma^{\rm pol}/d\mathbf{q})$ with the integrand taken from Eq. (22). The dashed line represents the limit imposed by energy-momentum conservation for $\varphi = 0$. (b) Same as (a) integrated over θ for different polariton energies $\hbar\omega_p$ [see color labels in (d)]. (c) Energy-integrated cross section $d\sigma^{\rm pol}/d\theta d\varphi = \sin\theta\int_0^\infty q^2 dq\,(d\sigma^{\rm pol}/d\mathbf{q})$ as a function of polar and azimuthal emission angles (θ,φ) under the conditions of (a). (d) Same as (c) integrated over φ for different polariton energies. (e) Total cross section [$\mathbf{q}$-integral of Eq. (22)] as a function of polariton wave vector k_p and energy $\hbar\omega_p$. For reference, we show the dispersion relations of free-space light ($\omega = ck$), p-polarized modes in a dielectric waveguide [10] (80 nm thickness, 2.24 refractive index), graphene plasmons (1 eV Fermi energy), and the A exciton in monolayer WS_2 [11].

for the small polariton-induced pair-production cross section at such relatively small γ-photon energies [e.g., $\sigma^{\rm pol} \sim 0.1$ pbarn for few-eV polaritons and 1.17 MeV γ-photons; see Figure 2(c) in the main text]. For example, considering again γ-photons delivered by a ^{60}Co source close to a polariton-supporting surface, we can have a flux of 10^{14} γ-photons/s cm^2, which, when multiplied by $\sigma^{\rm pol}$, by the number of polaritons $N_p \sim 10^{19}$, and by the polariton lifetime (e.g., nanoseconds for high-index planar dielectric waveguides with quality factors $\sim 10^6$), leads to $\sim 10^{-13}$ pairs per laser pulse, which can be collected over a time measurement window of ~ 0.1 ns using currently available fast electronics.

Considering the use of these kinds of sources, we consider $\hbar\omega_\gamma = 1.17$ MeV and compute the emitted positron distribution predicted by Eq. (22) as a function of kinetic energy $E_q^{\rm kin} = \hbar\varepsilon_q - m_e c^2$ and polar angle θ under the configuration depicted in Figure 2(a) of the main text. The result [Supplementary Figure **2**(a)] indicates a preference for polar angles close to normal when the positron takes most of the energy (electron emitted nearly at rest), and conversely, grazing emission for low-energy positrons. The spectral distribution obtained by further integrating over θ displays a symmetric profile with respect to the central peak found at $E_q^{\rm kin} = (\hbar\omega_\gamma - 2m_e c^2)/2 \approx 74$ keV [Supplementary Figure **2**(b)], as expected from the electron-positron kinematical symmetry. In addition, the energy-integrated positron-emission cross section is nearly independent of azimuthal angle φ [Supplementary Figure **2**(c)] because of the comparatively small in-plane momentum carried by the polaritons, while the polar dependence shows a maximum at around $\theta \sim 45°$, in good correspondence with the symmetrically arranged pair emission, dominated by the spectral maximum in Supplementary Figure **2**(b). Finally, the full $\mathbf{q}$-integrated cross section [Supplementary Figure **2**(e)] shows a nearly uniform increase with polariton frequency as $\propto \omega_p^2$, except for the depletion observed when k_p moves close to the light cone (dashed line). Overall, we conclude that the studied process leads to a strong angular and energy dependence of the resulting positron emission, which should facilitate an experimental verification of these results, although the background coming from Bethe-Heitler scattering at the polaritonic material imposes a severe constrain, as we discuss in the main text.

B. Pair production with GeV γ-photons

We analyze the emitted positron distribution in Supplementary Figure **3** for 1 GeV γ-photons, which can be experimentally produced by bremsstrahlung and Compton backscattering [15], while several proposals for more efficient sources have recently been put forward based on electron-beam collisions with intense laser spots

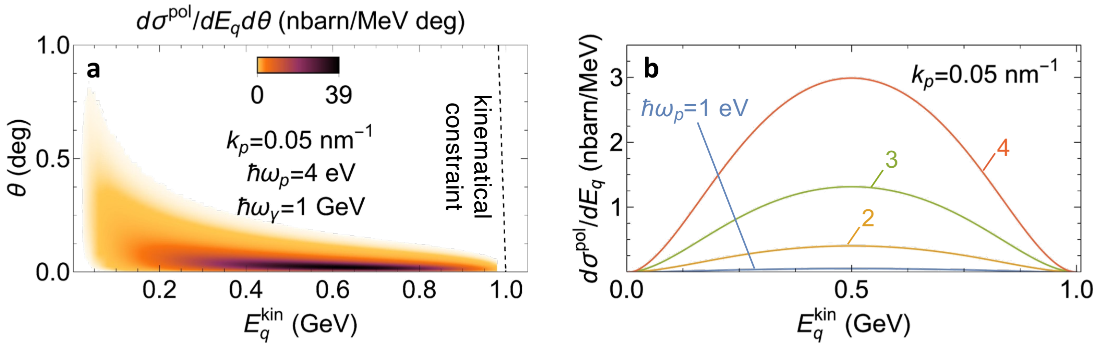

Supplementary Figure 3: **Pair production with polaritons and GeV γ-photons.** (a) Pair-production differential cross section as a function of positron polar angle θ and kinetic energy $E_q^{\rm kin} = \hbar\varepsilon_q - m_e c^2$ (normalized to the γ-photon energy $\hbar\omega_\gamma = 1$ GeV and averaged over an energy window $\Delta E_q^{\rm kin} = 1$ keV) for fixed polariton momentum $k_p = 0.05$ nm^{-1} and energy $\hbar\omega_p = 4$ eV. The dashed line represents the limit imposed by energy-momentum conservation for an azimuthal angle $\varphi = 0$. (b) Spectral distribution of positron emission (integrated over θ) for different polariton energies.

[16, 17], strong laser irradiation of electron plasma [18, 19], simultaneous laser and electron plasma bombardment [20], and electrons impinging on solid targets [21].

Upon integration of Eq. (22) over the azimuthal positron emission angle φ, Supplementary Figure **3**(a) illustrates how the differential cross section $d\sigma^{\rm pol}/dE_q d\theta$ [see Eq. (14)] is strongly peaked around normal emission (polar angle $\theta \sim 0$). In addition, positrons are preferentially sharing about half of the photon energy [Supplementary Figure **3**(b)], with a similar spectral distribution regardless of the polariton energy and a strong increase in emission efficiency with polariton energy $\hbar\omega_p$ [already observed in Supplementary Figure **2**(b) for 1.17 MeV γ-photons].

We remark that polaritonic modes can be strongly populated by irradiation with ultrafast laser pulses at fluences creating a surface polariton density as high as $\rho_p \sim 1/{\rm nm}^2$ without causing material damage, such that the scattering of 1 GeV photons [$\sigma^{\rm pol} \sim 10^{-6}$ barn; see Figure 2(c) in the main text] at a currently attainable rate $r_\gamma \sim 10^6$/s [22] would lead to a pair-production rate $\rho_p r_\gamma \sigma^{\rm pol} \sim 10^{-10}$/s, while higher rates could potentially be achieved with alternative designs for efficient GeV photon sources [16, 20].

Note 5. ADDITIONAL REMARKS

1. The BH cross section is [3] $\sigma^{\rm BH} = (e^2/\hbar c)(Ze^2/m_e c^2)^2\Big\{2x^2\big[2C_2(x) - D_2(x)\big] + (2/27)\big[(109 + 64x^2)[K(\xi) - E(\xi)] - (67 + 6x^2)(1 - x^2)K(\xi)\big]\Big\}$ where $C_2(x) = \int_1^{1/x} dy\, \cosh^{-1}(y)\cosh^{-1}(1/xy)/y$, $D_2(x) = \int_1^{1/x} dy\, \cosh^{-1}(1/xy)/\sqrt{y^2-1}$, $\xi = \sqrt{1-x^2}$, valid for $x = 2m_e c^2/\hbar\omega_g \leq 1$. Note that a minus sign in Ref. [3] is corrected in this expression by comparing it with Ref. [23]. The latter reference contains a different typo (a minus sign exchanged with a plus sign), which we also correct here. Upon numerical integration, the expression that we offer here reproduces the results tabulated in Ref. [24].

2. The positron escape depth can be estimated from the measured transmission through films of different thickness [25], which reveals a depletion down to 10% for a thickness of $\sim 5\,\mu$m in common metals including gold at 75 keV (i.e., the average kinetic energy per particle when producing pairs using the 1.17 MeV line of ^{60}Co).

3. The BW cross section is [3] $\sigma^{\rm BW} = (e^4\pi/2m_e^2c^4)(1-x^2)\big\{(3-x^4)\log[(1+x)/(1-x)] - 2x(2-x^2)\big\}$ with $x = \sqrt{1 - 2m_e^2c^4/[\hbar^2\omega_p\omega_g(1-\cos\theta_{p\gamma})]}$.

Supplementary References

[1] F. Mandl and G. Shaw, *Quantum Field Theory* (Wiley, Hoboken, 2010).

[2] Although the choice of a gauge with vanishing scalar potential can introduce complications related to overquantization of electromagnetic degrees of freedom (see, for example, Ref. [3]), such problem does not affect pair production by classical fields.

[3] J. M. Jauch and F. Rohrlich, *The Theory of Photons and Electrons* (Springer, Berlin, 1976).

[4] R. A. Álvarez-Puebla, L. M. Liz-Marzán, and F. J. García de Abajo, "Light concentration at the nanometer scale," J. Phys. Chem. Lett. **1**, 2428–2434 (2010).

[5] D. N. Basov, M. M. Fogler, and F. J. García de Abajo, "Polaritons in van der Waals materials," Science **354**, aag1992 (2016).
[6] Tony Low, Andrey Chaves, Joshua D. Caldwell, Anshuman Kumar, Nicholas X. Fang, Phaedon Avouris, Tony F. Heinz, Francisco Guinea, Luis Martin-Moreno, and Frank Koppens, "Polaritons in layered two-dimensional materials," Nat. Mater. **16**, 182–194 (2017).
[7] F. J. García de Abajo, "Graphene plasmonics: challenges and opportunities," ACS Photonics **1**, 135–152 (2014).
[8] J. D. Caldwell, I. Aharonovich, G. Cassabois, J. H. Edgar, B. Gil, and D. N. Basov, "Photonics with hexagonal boron nitride," Nat. Rev. Mater. **4**, 552–567 (2019).
[9] Z. M. Abd El-Fattah, V. Mkhitaryan, J. Brede, L. Fernández, C. Li, Q. Guo, A. Ghosh, A. Rodríguez Echarri, D. Naveh, F. Xia, J. E. Ortega, and F. J. García de Abajo, "Plasmonics in atomically thin crystalline silver films," ACS Nano **13**, 7771–7779 (2019).
[10] The dispersion relation of p-polarized guided modes in a self-standing dielectric waveguide with real refractive index $n=\sqrt{\epsilon}$ and thickness d is computed numerically by solving the equations $\cot(k_z d/2)=-\epsilon\kappa_z/k_z$ and $\tan(k_z d/2)=\epsilon\kappa_z/k_z$, with $k_z=\sqrt{\omega_p^2/c^2-k_p^2}$ and $\kappa_z=\sqrt{k_p^2-\omega_p^2\epsilon/c^2}$ [14].
[11] The quasistatic dispersion relation of a surface polariton propagating along a 2D material with conductivity $\sigma(\omega)=(\mathrm{i}e^2/\hbar)\,\omega_D/(\omega-\omega_g+\mathrm{i}\gamma)$ is $\omega_p=\omega_g/2+\sqrt{\omega_g^2/4+2\pi e^2\omega_D k_p\hbar}$, as obtained from $\omega_p=-2\pi\mathrm{i}\sigma k_p$ [7]. For graphene plasmons, we set $\omega_g=0$ and $\hbar\omega_D=E_F/\pi$, whereas for WS_2 excitons, we set $\hbar\omega_g=2$ eV and $\hbar\omega_D=100$ meV [26].
[12] A. Ansón-Casaos, J. A. Puértolas, F. J. Pascual, J. Hernández-Ferrer, P. Castell, A. M. Benito, W. K. Maser, and M. T. Martínez, "The effect of gamma-irradiation on few-layered graphene materials," Appl. Surf. Sci. **301**, 264–272 (2014).
[13] L. F. Dumée, C. Feng, L. He, F.-M. Allioux, Z. Yi, W. Gao, C. Banos, J. B. Davies, and L. Kong, "Tuning the grade of graphene: gamma ray irradiation of free-standing graphene oxide films in gaseous phase," Appl. Surf. Sci. **322**, 126–135 (2014).
[14] E. J. C. Dias and F. J. García de Abajo, "Complete coupling of focused light to surface polaritons," Optica **8**, 520–531 (2021).
[15] V. G. Nedorezov, A. A. Turinge, and Yu M. Shatunov, "Photonuclear experiments with Compton-backscattered gamma beams," Phys.-Uspekhi **47**, 341–358 (2004).
[16] A. Gonoskov, A. Bashinov, S. Bastrakov, E. Efimenko, A. Ilderton, A. Kim, M. Marklund, I. Meyerov, A. Muraviev, and A. Sergeev, "Ultrabright GeV photon source via controlled electromagnetic cascades in laser-dipole waves," Phys. Rev. X **7**, 041003 (2017).
[17] J. Magnusson, A. Gonoskov, M. Marklund, T. Zh. Esirkepov, J. K. Koga, K. Kondo, M. Kando, S. V. Bulanov, G. Korn, and S. S. Bulanov, "Laser-particle collider for multi-GeV photon production," Phys. Rev. Lett. **122**, 254801 (2019).
[18] Chen Liu, Baifei Shen, Xiaomei Zhang, Liangliang Ji, Zhigang Bu, Wenpeng Wang, Longqing Yi, Lingang Zhang, Jiancai Xu, Tongjun Xu, , and Zhikun Pei, "Ultra-bright, well-collimated, GeV gamma-ray production in the QED regime," Phys. Plasmas **25**, 023107 (2018).
[19] Xing-Long Zhu, Tong-Pu Yu, Min Chen, Su-Ming Weng, , and Zheng-Ming Sheng, "Generation of GeV positron and γ-photon beams with controllable angular momentum by intense lasers," New J. Phys. **20**, 083013 (2018).
[20] Xing-Long Zhu, Min Chen, Su-Ming Weng, Tong-Pu Yu, Wei-Min Wang, Feng He, Zheng-Ming Sheng, Paul McKenna, Dino A. Jaroszynski, and Jie Zhang, "Extremely brilliant GeV γ-rays from a two-stage laser-plasma accelerator," Sci. Adv. **6**, eaaz7240 (2020).
[21] Archana Sampath and others, "Extremely dense gamma-ray pulses in electron beam-multifoil collisions," Phys. Rev. Lett. **126**, 064801 (2021).
[22] N. Muramatsu and others, "SPring-8 LEPS2 beamline: A facility to produce a multi-gev photon beam via laser Compton scattering," Nucl. Instrum. Methods Phys. Res. A **1033**, 166677 (2022).
[23] R. Jost, J. M. Luttinger, and M. Slotnick, "Distribution of recoil nucleus in pair production by photons," Phys. Rev. **80**, 189–196 (1950).
[24] L. C. Maximon, "Simple analytic expressions for the total Born approximation cross section for pair production in a Coulomb field," J. Res. Natl. Bur. Stand. **72B**, 79–88 (1968).
[25] H. H. Seliger, "Transmission of positrons and electrons," Phys. Rev. **15**, 1029–1037 (1955).
[26] Yilei Li, Alexey Chernikov, Xian Zhang, Albert Rigosi, Heather M. Hill, Arend M. van der Zande, Daniel A. Chenet, En-Min Shih, James Hone, and Tony F. Heinz, "Measurement of the optical dielectric function of monolayer transition-metal dichalcogenides: MoS_2, $MoSe_2$, WS_2, and WSe_2," Phys. Rev. B **90**, 205422 (2014).
